# Mr. Gladstone: Sir George Stokes

**Published:** 1891-01-03

**Authors:** 


### MR. GLADSTONE: SIR GEORGE STOKES.

Two very eminent persons outside the medical profession made incursions into regions which, though not both medical, yet have a profound interest for physicians and pure physiologists. Mr. Gladstone visited Guy's Hospital and formally opened the Students' House. In an audience bristling with medical persons of all sorts, he frankly confessed that he himself had practised unlicensed medical treatment, and that it had been singularly successful. Wielding his woodman's axe one day in the dim and trackless forest of Hawarden, he had the misfortune to inflict upon himself a somewhat severe wound. The blood flowed freely; and, as neither Sir Andrew Clark nor the Hawarden Æsculapius was immediately available, Mr. Gladstone applied some of the leaves of a neighbouring tree to the staunching of the wound, and with immediate success. The audience assembled at Guy's did not consult the solicitor of the Medical Defence Association. The unlicensed practitioner was permitted to escape for that time. Sir George Stokes, the distinguished president of the Royal Society, appealed to the profoundest imaginations and the grandest hopes of practically all the members of the human race when he expressed his belief in the possibility of a future life. This is not the place to give even an outline of his argument. But that so distinguished a representative of all that is profoundest and best in the science of the times should have announced his belief in such a possibility is not only an encouragement to the philosophical moralist, but a distinct indication that the reign of the "scientific mechanic" is coming to an end, and that science herself will soon become as philosophical, and as full of poetry and life as she has recently been wooden and cramped and dead.

